# Multi-Objective Optimization Accelerates the De Novo Design of Antimicrobial Peptide for *Staphylococcus aureus*

**DOI:** 10.3390/ijms252413688

**Published:** 2024-12-21

**Authors:** Cheng-Hong Yang, Yi-Ling Chen, Tin-Ho Cheung, Li-Yeh Chuang

**Affiliations:** 1 Department of Electronic Engineering, National Kaohsiung University of Science and Technology, Kaohsiung 807618, Taiwan; chyang@nkust.edu.tw (C.-H.Y.); yiling100328@gmail.com (Y.-L.C.); f112152195@nkust.edu.tw (T.-H.C.); 2 Department of Information Management, Tainan University of Technology, Tainan 710302, Taiwan; 3 Ph.D. Program in Biomedical Engineering, Kaohsiung Medical University, Kaohsiung 807378, Taiwan; 4 Drug Development and Value Creation Research Centre, Kaohsiung Medical University, Kaohsiung 807378, Taiwan; 5 Department of Chemical Engineering & Institute of Biotechnology Engineering and Chemical Engineering, I-Shou University, Kaohsiung 824005, Taiwan

**Keywords:** multi-objective optimization, physicochemical properties, antimicrobial peptide, *Staphylococcus aureus*

## Abstract

Humans have long used antibiotics to fight bacteria, but increasing drug resistance has reduced their effectiveness. Antimicrobial peptides (AMPs) are a promising alternative with natural broad-spectrum activity against bacteria and viruses. However, their instability and hemolysis limit their medical use, making the design and improvement of AMPs a key research focus. Designing antimicrobial peptides with multiple desired properties using machine learning is still challenging, especially with limited data. This study utilized a multi-objective optimization method, the non-dominated sorting genetic algorithm II (NSGA-II), to enhance the physicochemical properties of peptide sequences and identify those with improved antimicrobial activity. Combining NSGA-II with neural networks, the approach efficiently identified promising AMP candidates and accurately predicted their antibacterial effectiveness. This method significantly advances by optimizing factors like hydrophobicity, instability index, and aliphatic index to improve peptide stability. It offers a more efficient way to address the limitations of AMPs, paving the way for the development of safer and more effective antimicrobial treatments.

## 1. Introduction

Antibiotics have revolutionized medicine, becoming a cornerstone of treatment for bacterial infections. They can kill bacteria by disrupting their cell walls or inhibit their growth by interfering with essential processes. However, the overuse of antibiotics has led to a concerning rise in multi-drug-resistant (MDR) bacterial infections [1]. *Staphylococcus aureus* (SA) is one of the most prevalent MDR pathogens and is the leading bacterial cause of death in 135 countries, with the highest mortality rate among individuals over 15 years old globally [2]. These bacteria have developed mechanisms to resist commonly used antibiotics, creating significant challenges for infection treatment and control.

Antimicrobial peptides (AMPs) are a unique and diverse group of molecules, typically 12 to 50 amino acids. Produced by various organisms, from insects to humans, they play a crucial role in innate immunity [3]. AMPs demonstrate broad-spectrum activity, killing Gram-positive and Gram-negative bacteria, enveloped viruses, fungi, and even transformed or cancerous cells [4]. Unlike conventional antibiotics, AMPs employ various mechanisms of action, including disrupting cell membrane integrity and causing perforation, as well as potentially interfering with protein synthesis or inhibiting essential enzymes [4]. However, these promising candidates often have deficiencies, such as instability, short half-life, severe hemolytic activity, and susceptibility to proteolytic degradation [5]. Therefore, designing new AMPs with improved stability, longer half-lives, reduced side effects, and resistance to proteolytic degradation is an urgent issue.

Although AMPs offer significant promise for therapeutic applications, their transition to clinical use has been hindered by practical challenges [6]. One major obstacle is their limited bioavailability, as absorption is significantly reduced by stomach acid, liver metabolism, kidney filtration, and other digestive processes [7,8,9]. Furthermore, AMPs are often rapidly cleared from the bloodstream, which limits their therapeutic efficacy. Interactions with unintended targets or receptors can lead to side effects, including immunogenic reactions [10]. Additionally, while the structural and functional diversity of AMPs provides opportunities for generating numerous peptide sequences, it also complicates the design process. To overcome these challenges, it is crucial to redesign and optimize AMPs to achieve enhanced stability, efficacy, and safety while maintaining broad-spectrum activity [11].

Machine learning (ML), a subset of artificial intelligence, has become an essential tool in medical research for addressing complex and multifaceted problems. In recent years, various computational models with distinct predictive mechanisms have been applied to peptide sequences [12]. These computational methods are increasingly valuable in antimicrobial peptide (AMP) research. Researchers frequently use ML models such as Support Vector Machines (SVM), Logistic Regression (LR), Random Forest (RF), K-Nearest Neighbor (KNN), and Neural Networks to perform binary classification, categorizing peptides as either “AMP” or “non-AMP” [13,14,15,16]. Feature selection is sometimes employed to identify relevant physicochemical properties for inclusion in ML models [17,18]. A key advantage of these computational techniques is their ability to explore unknown peptide sequences, broadening the scope for discovery and innovation in AMP design and screening.

In this study, we aim to discover and engineer novel AMPs that exhibit enhanced efficacy against SA. To achieve this, the Database of Antimicrobial Activity and Structure of Peptides (DBAASP) (https://dbaasp.org/search, accessed on 28 February 2024) [19] was used to identify potential peptide candidates for further investigation. We employed a powerful multi-objective optimization technique, the non-dominated sorting genetic algorithm II (NSGA-II), to design AMPs. The candidate sequences were evaluated based on three essential physicochemical properties, including hydrophobicity, instability index, and aliphatic index, critical for effective AMPs against SA. By integrating these parameters within the NSGA-II framework, we systematically explore large sequence spaces to identify AMPs with optimized antimicrobial characteristics against SA. By applying these constraints and an exhaustive search strategy, all possible peptide sequences were comprehensively examined in a computational manner, thus facilitating the discovery of AMPs with enhanced antimicrobial properties against SA.

## 2. Results

### 2.1. Multi-Objective Optimization Result

The NSGA-II explores constraints similar to those: hydrophobicity (gravy) greater than 0, instability index smaller than 40, and aliphatic index greater than 71. These constraints ensure that the generated AMPs possess the desired physicochemical properties. The genetic algorithm (GA) type and important parameter specifications used in this experimental research are defined in Table 1 below.

**Figure 1:**
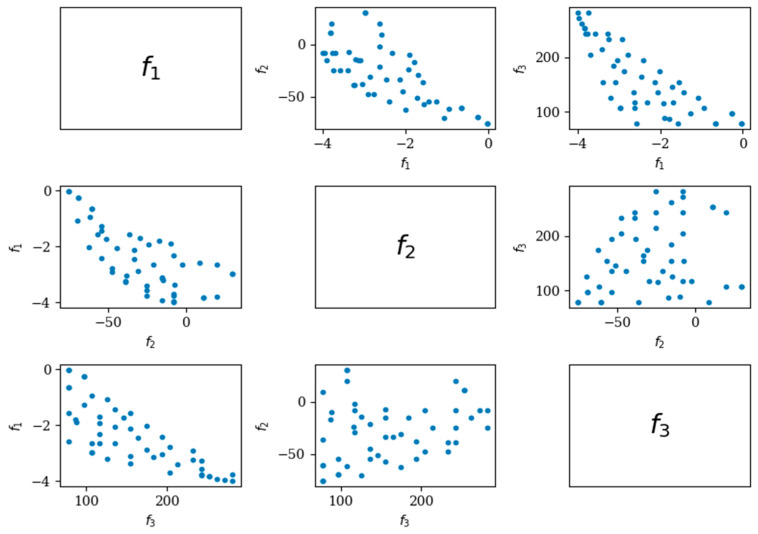
The final non-dominate results of the three physicochemical properties. f1 is the hydrophobicity (gravy); f2 is the instability index; f3 is the aliphatic index.

**Figure 2:**
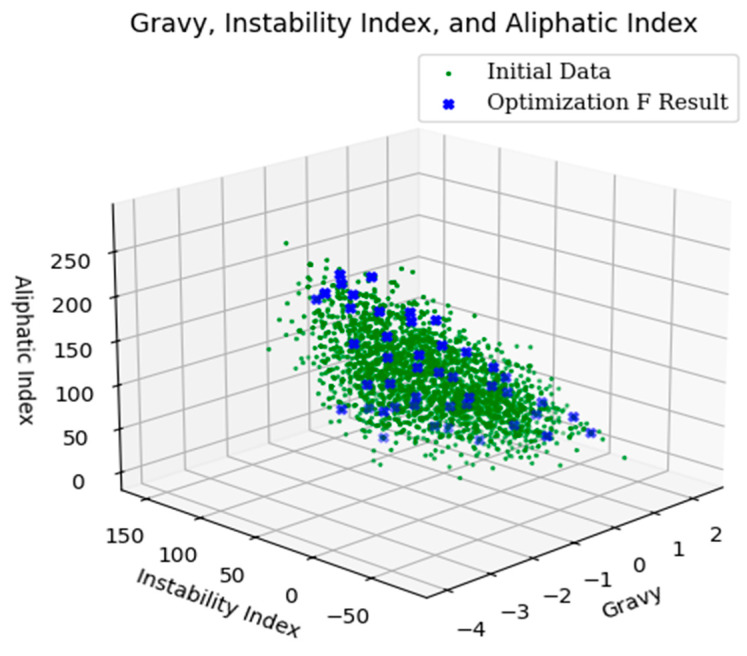
NSGA-II searching space.

The 5000 iterations of the search, as shown in Figure 1 and Figure 2, illustrate the NSGA-II search results and space. Due to the program’s requirements, each objective function must be minimized, and each constraint must be provided in ≤0 [20]. As a result, in Figure 1 and Figure 2, the gravy score is inverted: <0 indicates hydrophobicity, and >0 indicates hydrophilicity.

### 2.2. Neural Networks Result

**Figure 3:**
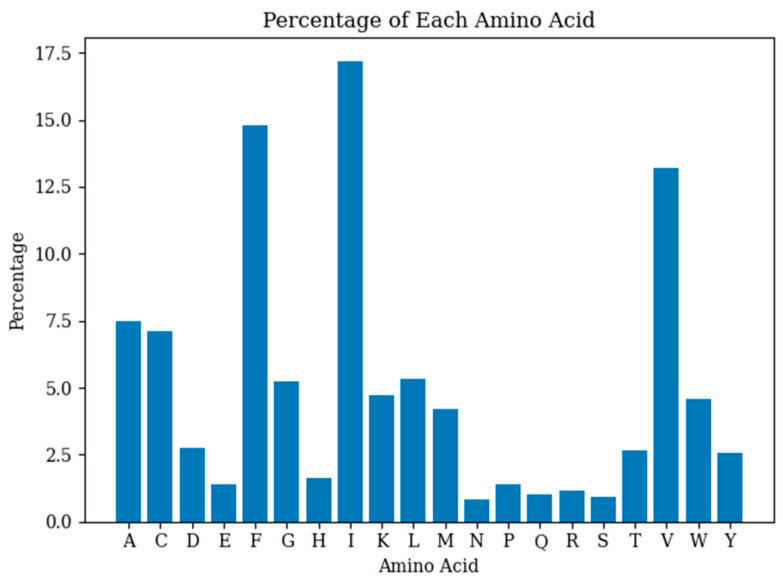
The percentage of each amino acid in the new generated sequences.

To develop a model for predicting the MIC values of antimicrobial peptides, this study trained a GRU on 1448 samples, utilizing bootstrap sampling for repeated training data extraction. The trained model assessed the antimicrobial peptide sequences identified by NSGA-II. Table 2 shows the NSGA-II search results using the GRU for predicting MIC values, while Figure 3 illustrates the percentage distribution of each amino acid in the sequences generated by NSGA-II. An experimental assay further verified the MIC prediction results.

### 2.3. Antimicrobial Peptides Design Metrics

Peptides, chains of amino acids, play a crucial role in various biological processes. These amino acids, represented by a one-letter code within a 20-letter alphabet, possess unique physicochemical properties that significantly influence their function. Each amino acid has characteristics such as hydrophobicity (gravy), charge (acidic or basic), and other relevant attributes. Our primary focus is the three essential physicochemical properties: hydrophobicity, instability index, and aliphatic index.

Hydrophobicity: This measures the relative hydrophobicity of the amino acids in the peptide sequence. Hydrophobicity significantly influences how the peptide interacts with lipid membranes. Hydrophobic amino acids aid in inserting and disrupting bacterial membranes, a crucial mechanism for antimicrobial activity. A higher hydrophobicity often correlates with enhanced membrane interaction and disruption, leading to increased antimicrobial effectiveness [21]. Hydrophobicity can be calculated as follows:

(1) $$Hydrophobicity=\frac{1}{l}\sum_{i=1}^{l}x_{i}$$

where l is the length of the peptide sequence and $x_{i}$ is the hydrophobicity value for each amino acid according to the Kyte–Doolittle scale.

Instability Index: This is a measure of protein stability. Peptides with a low instability index are more likely to be stable and resist degradation, making them more effective as antimicrobial agents. The instability index is defined using the weight values assigned to each of the 400 different dipeptides (DIWV) [22]. The formula for computing the instability index is written as follows:

(2) $$Instability\;Index=\left(\frac{l}{10}\right)\sum_{i=1}^{l-1}DIWV(dipeptide(i))$$

where l is the length of the peptide sequence and DIWV(dipeptide(i)) is the instability weight value for the dipeptide starting at position (i).

Aliphatic Index: This measures the relative volume of aliphatic side chains (alanine, valine, isoleucine, and leucine) in the peptide. A higher aliphatic index is often associated with increased thermostability, which can enhance the peptide’s functional lifespan in various environments [23]. The aliphatic index can be calculated as follows:

(3) $$Aliphatic\;Index=1\left(A\%\right)+2.9\left(V\%\right)+3.9(I\%+L\%)$$

where *A*%, *V*%, *I*%, and *L*% represent the percentage of alanine, valine, isoleucine, and leucine residues in the peptide sequence, respectively.

### 2.4. Antibacterial Activity of the Designed Peptides

The minimum inhibitory concentration (MIC) of the designed antimicrobial peptides (AMPs) was assessed using the microdilution method. The results indicated that all the designed AMP peptides displayed antibacterial activity against the tested strains of *Staphylococcus aureus*. Some peptides even demonstrated higher activity than clinically used antimicrobial peptides (colistin, polymyxin B, and poly-L-lysine hydrobromide) and antibiotics (tetracycline). Peptide 11 demonstrated significantly higher antimicrobial activity against clinical multidrug-resistant isolates. However, the inhibitory effects varied across different bacterial strains, suggesting that there is potential for enhancing broad-spectrum antimicrobial efficacy. A summary of the evaluation results for antimicrobial activity is presented in Table 3.

## 3. Discussion

The emergence of antimicrobial resistance (AMR) poses a significant challenge to global health, requiring exploring new therapeutic approaches. Among these options, antimicrobial peptides (AMPs) have attracted substantial attention. Despite their potential, AMPs encounter several barriers that hinder their widespread adoption.

### 3.1. Key Limitations of AMPs

Key limitations of antimicrobial peptides (AMPs) include inadequate targeting within the body, potential toxicity, hemolytic activity, rapid enzymatic degradation, and other adverse effects [5]. Furthermore, bacterial resistance to AMPs is rising, resembling the trend observed with conventional antibiotics. Bacteria use various mechanisms to evade the antimicrobial effects of peptides, such as proteolytic degradation, membrane modification, uptake transporters, and efflux pumps [24]. These resistance mechanisms reduce the effectiveness of AMPs, especially when used as a standalone treatment. It is crucial to address these challenges to fully leverage the therapeutic potential of AMPs in combating antimicrobial resistance.

### 3.2. Considered Physicochemical Properties

Throughout the paper, we focused only on three physicochemical properties critical for designing effective AMPs against SA: hydrophobicity, instability index, and aliphatic index. While these properties form the foundation of our approach, it is important to acknowledge that other factors also influence AMP efficacy, such as net charge, peptide length, and secondary structure composition [25].

Cationic AMPs are electrostatically attracted to negatively charged components of microbial membranes. Increasing the net positive charge of these peptides enhances their interaction with bacterial membranes, promoting aggregation and enabling them to reach a threshold concentration necessary for membrane rupture [26]. The primary mechanism of action for most AMPs involves disrupting the lipid bilayer through pore formation, ultimately leading to membrane integrity loss [27]. In this process, the cationic region of AMPs initiates membrane contact, while the hydrophobic region facilitates their transport across the lipid bilayer. These amphipathic peptides, often disordered in aqueous solutions, adopt active secondary structures—such as α-helices, β-turns, or β-sheets—when interacting with the lipid environment [28,29].

Moreover, the specific amino acid composition of AMPs can confer unique properties, such as increased resistance to proteolytic degradation, enhanced cell selectivity, or improved targeting of specific bacterial species [30]. Therefore, a robust foundation for designing antimicrobial peptides can be established by considering hydrophobicity, instability index, and aliphatic index. Considering a broader range of physicochemical properties can further improve and optimize the effectiveness and selectivity of AMPs against Staphylococcus aureus and other pathogens.

### 3.3. MIC Prediction

In Table 2, we generated multiple AMP sequences and utilized the GRU to forecast their MIC. In this primary study, we relied on the AMP scanner [31] predictions to evaluate the likelihood of these sequences being antimicrobial, as depicted in Table 4. This approach provides an additional layer of verification for our computational predictions. From an initial pool of predicted AMPs, the AMP scanner identified nine sequences with a high probability of an AMP being active against Gram-positive and/or Gram-negative bacteria. We randomly selected nine peptides predicted to be AMPs and three peptides predicted to be non-AMPs for activity evaluation. A comparison of the experimental results (Table 3) with the predictions revealed some discrepancies in MIC values, likely due to the diversity of the tested bacterial strains. Nonetheless, the overall findings confirm that the nine predicted AMP peptides exhibited antibacterial activity. In contrast, the non-AMP peptides (peptides 6–8) did not show significant antibacterial activity against any tested bacterial strains.

### 3.4. Techniques Application

This study employs multi-objective optimization and machine learning techniques to predict the minimum inhibitory concentration (MIC) values and determine whether peptides are AMP or non-AMP. Additionally, it forecasts their inhibitory concentration and hemolytic activity. The proposed method can help scientists swiftly identify peptide sequences with high antibacterial activity, thus accelerating the development of new antibacterial drugs.

### 3.5. Future Directions in AMP Optimization

Future studies could explore the impact of additional physicochemical properties and key factors on creating even more powerful AMPs. Additionally, it is important to recognize that NSGA-II, a fundamental algorithm for multi-objective optimization, was introduced more than 20 years ago. Advancements in optimization algorithms have since been made, such as multi-objective particle swarm optimization (MOPSO), UNSGA-III, and multi-objective Harris hawks optimization (MOHHO) [32]. Incorporating new algorithms into the design process could improve the efficiency and effectiveness of identifying optimal AMP sequences with desired physicochemical properties. Therefore, future research could explore applying these advanced optimization methods to further refine the design and development of AMPs for combating antimicrobial resistance.

## 4. Materials and Methods

To explore the AMP design space for targeting SA, we leverage the DBAASP database to acquire relevant AMP sequences. These sequences are a foundation for generating novel AMP candidates using the NSGA-II. This multi-objective optimization approach allows us to balance multiple design objectives simultaneously. Subsequently, a neural network model is employed to predict the minimum inhibitory concentration (MIC) values for the newly generated AMP sequences.

### 4.1. Proposed Model

**Figure 4:**
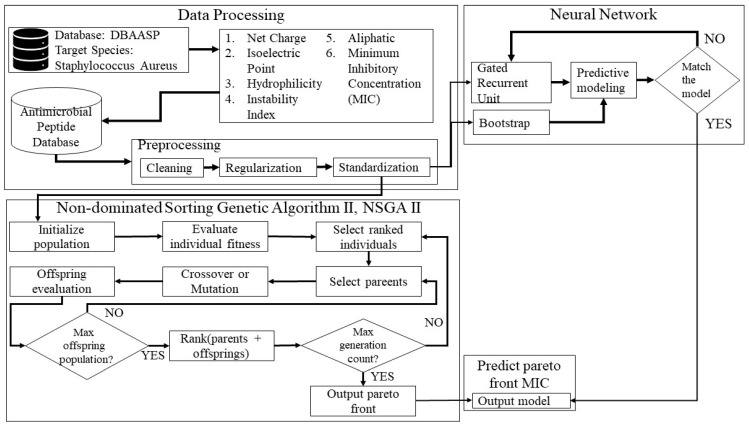
AMP scanner prediction for antimicrobial sequences using an algorithm for synthetic AMPs.

As illustrated in Figure 4, we proposed a comprehensive design model for generating and evaluating AMPs, which includes the following key components: (1) data processing, (2) multi-objective optimization, and (3) a neural network prediction model.

### 4.2. Dataset

In this study, we utilized the DBAASP, which contains 22,205 peptide sequences, categorized into 13 classes: Gram-positive (Gram+), Gram-negative (Gram−), virus, parasite, insect, cancer, fungus, mammalian cell, mollicute, nematode, protista, biofilm, and archaea. There are 1627 peptide sequences specifically targeting SA.

### 4.3. Methods

#### 4.3.1. Non-Dominated Sorting Genetic Algorithm II

**Figure 5:**
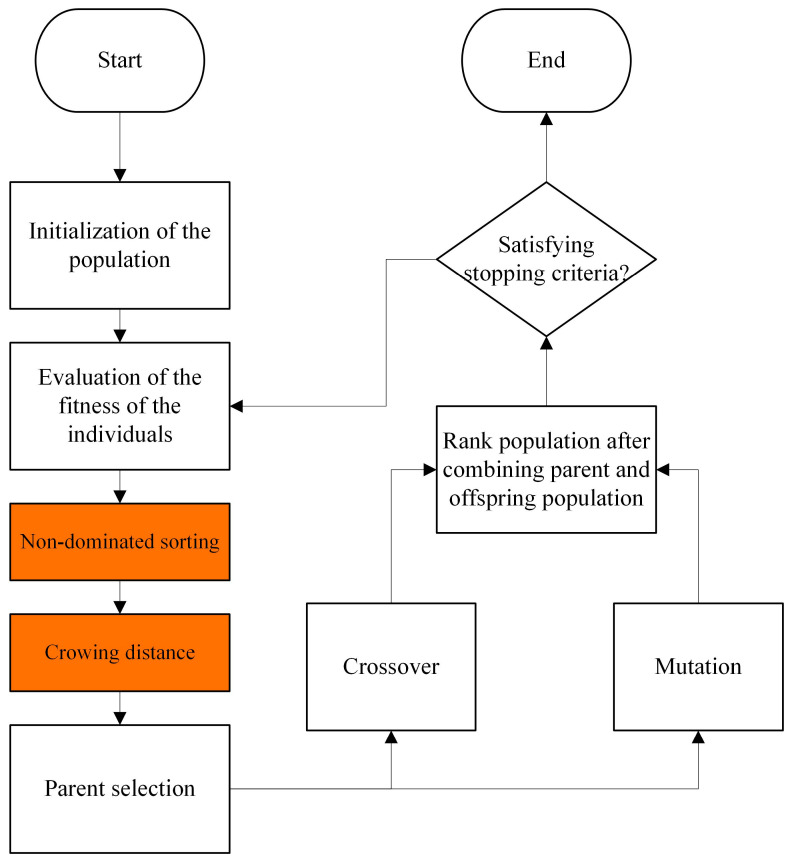
NSGA-II structure.

The NSGA-II [33] is a typical multi-objective optimization algorithm known for its well-established reputation in achieving stable and reliable results in complex problems with multiple, often conflicting, objectives. The NSGA-II structure is shown as Figure 5. This is particularly beneficial for AMP design, where we must balance crucial physicochemical properties such as hydrophobicity, instability index, and aliphatic index to achieve optimal potency. NSGA-II excels at handling these trade-offs by efficiently exploring the vast sequence space and identifying AMPs that exhibit high efficacy against SA while maintaining desirable stability and reduced side effects. Compared to traditional optimization methods, NSGA-II’s ability to identify diverse, non-dominated solutions holds promise for discovering novel AMPs with a broader range of beneficial properties [34].

The multi-objective problem (MOP) that models the SA design problem is defined as follows:

(4) $$\begin{array}{c} max\;f_{1}\left(X\right)=Hydrophobicity\\ min\;f_{2}\left(X\right)=Instability\;Index\\ min\;f_{3}\left(X\right)=Aliphatic\;Index \end{array}$$

subject to the following:

$$f_{1}\left(X\right)>0\;f_{2}\left(X\right)<40\;f_{3}\left(X\right)>71$$

In this equation, we set constraints to limit peptides to a specific range of hydrophobicity, stability, and a higher aliphatic index. These constraints ensure that the selected AMPs exhibit optimal antimicrobial activity and stability performance.

The constraint $f_{1}\left(X\right)>0$ within a specified range indicates that the peptide is effective in interacting with bacterial membranes, which is crucial for its antimicrobial activity. This constraint ensures that the peptide has the necessary hydrophobicity to interact with the bacterial membrane, a key aspect of its mechanism of action against microbes.

The constraint $f_{2}\left(X\right)<40$ ensures that the peptide is stable and resistant to degradation. Stability is critical for the practical application of AMPs, as they must withstand various environmental conditions and enzymatic degradation in biological systems.

The constraint $f_{3}\left(X\right)>71$ contributes to the peptide’s thermostability and overall robustness. A higher aliphatic index is associated with increased thermostability, making the peptide more resilient to temperature variations and environmental stresses.

#### 4.3.2. Neural Networks

**Figure 6:**
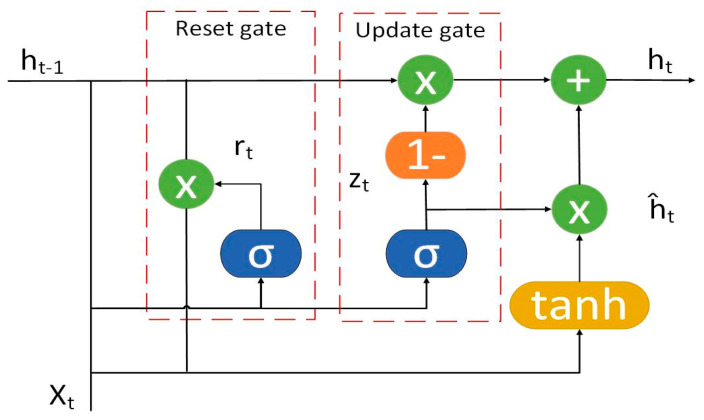
GRU network structure.

The gated recurrent unit (GRU) is a type of recurrent neural network designed to manage the flow of information through its architecture using reset and update gates. At each time step, the GRU takes an input vector and a hidden state vector, and it outputs a new hidden state vector along with an output vector. The hidden state essentially signals whether to fully retain (‘1’) or forget (‘0’) the information from the previous hidden state. The update gate controls how much past information is brought forward to the current hidden state, while the reset gate determines which part of the previous hidden state should be forgotten and how much of the current input should be highlighted. GRUs learn through backpropagation, adjusting weight matrices and activation functions during training. The sigmoid activation function is employed for both the update and reset gates, whereas the tanh activation function is used for computing the current hidden state. Compared to long short-term memory (LSTM) networks, GRUs are simpler and involve fewer parameters, making them faster and more memory-efficient to train. The GRU-important parameter specifications used in this experimental research are defined, and the network structure is shown in Figure 6 and Table 5 below.

### 4.4. Evaluation of Antibacterial Activity

The antibacterial activity was evaluated using a microdilution method [35]. We randomly selected 12 peptides from the designed set for evaluation. The test included *Staphylococcus aureus* strains consisting of two clinical multidrug-resistant isolates (Sa 1075 and Sa 2803) and a standard strain (Sa ATCC 6538P). In brief, 100 μL of Mueller–Hinton broth (MHB) was added to each well of a 96-well microplate. The peptide concentrations were prepared through serial dilutions ranging from 0.125 to 100 μg/mL. Positive controls, which included colistin, polymyxin B, poly-L-lysine hydrobromide, and the antibiotic tetracycline, were prepared in separate wells, each in triplicate. Subsequently, 10 μL of a 1:20 dilution of a 0.5 McFarland standard bacterial suspension (equivalent to 1 × 10^8^ CFU/mL) was added to each well, resulting in a final concentration of 5 × 10^6^ CFU/mL. The plates were incubated at 35 ± 2 °C for 24 h. After the incubation period, bacterial growth was assessed by measuring the absorbance at 600 nm. The minimum inhibitory concentration (MIC) is defined as the lowest concentration at which the absorbance is less than half that of the sample without peptide.

## 5. Conclusions

This study combines NSGA-II and neural networks to develop a machine learning-based approach to drug design. The goal is to design new AMP sequences by utilizing numerical representations of the physicochemical properties of amino acids. NSGA-II is used to explore the physicochemical property space of AMPs, identifying sequences with potential antimicrobial activity. These sequences are then assessed using neural networks to predict their minimum inhibitory concentration. This combined approach identified promising AMP candidates and efficiently predicted their antibacterial activity, significantly reducing the time and resources that AMP developers typically waste on trial-and-error processes with uncertain results. Although the NSGA-II results may not always yield the absolute best candidates for AMP development, it serves as a valuable method for generating new AMP sequences with high potential. By optimizing multiple objectives, NSGA-II provides a systematic approach that can guide researchers in discovering novel AMPs that might otherwise go unnoticed, streamlining the design process and enhancing the likelihood of success in future drug development. The combination of NSGA-II and neural networks presents a new approach for speeding up the identification and improvement of AMPs. This could potentially result in the creation of new and powerful antimicrobial substances. Additionally, the method’s capability to effectively explore the intricate design possibilities of AMPs and forecast their effectiveness demonstrates its potential for wider applications in drug discovery and development.

## Figures and Tables

**Table 1:** Parameter setting of NSGA-II.

Population size	100
Number of iterations	50
Crossover probability	0.9
Mutation probability	0.9
Crossover index	15
Mutation index	20

**Table 2:** Examples of SA identified from NSGA-II.

Sequence	Gravy	Instability Index	Aliphatic Index	Isoelectric Point	Net Charge	MIC
CVVRCRCVFR	0.94	−6.08	87	9.307123	3	3.732
CVVVDRIVDR	0.78	−52.17	155	5.954078	0	6.47
RCVCCRIVRF	0.97	−7.03	97	9.307123	3	3.822
RCVCVRIVTR	0.79	−23.06	126	10.41282	3	4.727
RCVTRVVIVR	0.96	−15.52	155	11.70032	3	5.3
RCVVTRIVRF	0.82	−15.52	126	11.70032	3	4.797
RIVDRIVVVR	0.88	−30.55	194	11.69903	2	6.11
RIVDRVIVVR	0.88	−30.55	194	11.69903	2	6.11
RIVDRVVIVR	0.88	−30.55	194	11.69903	2	6.11
RIVVDRIVVR	0.88	−30.55	194	11.69903	2	6.11
RIVVDRVIVR	0.88	−30.55	194	11.69903	2	6.11
VCVCVRMRCR	0.85	−13.62	87	9.305834	3	4.19
VCVDRFVDRV	0.61	−43.68	116	5.92526	0	4.805
VCVDRVVDRF	0.61	−43.68	116	5.92526	0	4.805
VCVRCRCVFR	0.94	−6.08	87	9.305834	3	3.736
VCVVDRIVDR	0.78	−52.17	155	5.92526	0	6.473
VRVRIVYKVC	0.96	−15.52	155	10.05334	3	5.426
VRVTRCVIVR	0.96	−15.52	155	11.70032	3	5.29
VVDRCVDRIV	0.78	−52.17	155	5.92526	0	6.473

**Table 3:** Results of the microdilution test to determine the MIC of the peptide against various bacteria.

Peptides	MIC * (μg/mL)			
	Bacteria Strains			Predicted
	Sa 6538P	Sa 1075	Sa 2803	
1. CVVRCRCVFR	12.5	>100	>100	3.732
2. RCVCCRIVRF	12.5	>100	100	3.822
3. RCVCVRIVTR	100	>100	>100	4.727
4. RCVTRVVIVR	12.5	>100	>100	5.300
5. RCVVTRIVRF	12.5	>100	>100	4.797
6. RIVDRIVVVR	>100	>100	>100	6.110
7. RIVDRVIVVR	>100	>100	>100	6.110
8. RIVDRVVIVR	>100	>100	>100	6.110
9. VCVCVRMRCR	>100	>100	25	6.110
10. VCVRCRCVFR	>100	>100	12.5	4.805
11. VRVRIVYKVC	>100	12.5	3.125	6.473
12. VRVTRCVIVR	>100	>100	6.25	5.426
colistin	>100	>100	1.56	ND
polymyxin B	100	>100	12.5	ND
poly-L-lysine hydrobromide	50	100	50	ND
Tetracycline	1250	1250	1250	ND

Abbreviation: * MIC, minimum inhibitory concentration; ND, not determined.

**Table 4:** AMP scanner predictions for antimicrobial sequences using an algorithm for synthetic AMPs.

Sequence	Class	Probability	Sequence	Class	Probability
CVVRCRCVFR	AMP	0.9989	RIVVDRVIVR	Non-AMP	0.0283
CVVVDRIVDR	Non-AMP	0.0129	VCVCVRMRCR	AMP	0.9996
RCVCCRIVRF	AMP	0.9469	VCVDRFVDRV	Non-AMP	0.0268
RCVCVRIVTR	AMP	0.9947	VCVDRVVDRF	Non-AMP	0.089
RCVTRVVIVR	AMP	0.9385	VCVRCRCVFR	AMP	0.9916
RCVVTRIVRF	AMP	0.9885	VCVVDRIVDR	Non-AMP	0.0257
RIVDRIVVVR	Non-AMP	0.0643	VRVRIVYKVC	AMP	0.997
RIVDRVIVVR	Non-AMP	0.0699	VRVTRCVIVR	AMP	0.7774
RIVDRVVIVR	Non-AMP	0.0818	VVDRCVDRIV	Non-AMP	0.0085
RIVVDRIVVR	Non-AMP	0.0103			

AMP: antimicrobial; Non-AMP: non-antimicrobial. Note: Peptide is predicted to be antimicrobial if the probability is ≥0.5.

**Table 5:** Parameter setting of GRU.

Epochs	2000
Batch size	1024
Hidden layer activation	Linear
Layer	7

## Data Availability

The data used to support the findings of this study are included within the article.
